# Monocytes Contribute to Differential Immune Pressure on R5 versus X4 HIV through the Adipocytokine Visfatin/NAMPT

**DOI:** 10.1371/journal.pone.0035074

**Published:** 2012-04-06

**Authors:** Rafael Van den Bergh, Sébastien Morin, Hans Jürgen Sass, Stephan Grzesiek, Marc Vekemans, Eric Florence, Huyen Thanh Thi Tran, Rosina Gabriel Imiru, Leo Heyndrickx, Guido Vanham, Patrick De Baetselier, Geert Raes

**Affiliations:** 1 Department of Molecular and Cellular Interactions, VIB, Brussels, Belgium; 2 Laboratory of Cellular and Molecular Immunology, Vrije Universiteit Brussel, Brussels, Belgium; 3 Division of Structural Biology, Biozentrum der Universität Basel, Basel, Switzerland; 4 HIV/STD Unit, Department of Clinical Sciences, Institute of Tropical Medicine, Antwerp, Belgium; 5 HIV Virology Unit, Department of Microbiology, Institute of Tropical Medicine, Antwerp, Belgium; 6 Department of Biomedical Sciences, Faculty of Pharmaceutical, Biomedical and Veterinary Sciences, University of Antwerp, Antwerp, and Faculty of Medicine and Pharmacy, Vrije Universiteit Brussel, Brussels, Belgium; Academic Medical Center, The Netherlands

## Abstract

**Background:**

The immune system exerts a diversifying selection pressure on HIV through cellular, humoral and innate mechanisms. This pressure drives viral evolution throughout infection. A better understanding of the natural immune pressure on the virus during infection is warranted, given the clinical interest in eliciting and sustaining an immune response to HIV which can help to control the infection. We undertook to evaluate the potential of the novel HIV-induced, monocyte-derived factor visfatin to modulate viral infection, as part of the innate immune pressure on viral populations.

**Results:**

We show that visfatin is capable of selectively inhibiting infection by R5 HIV strains in macrophages and resting PBMC in vitro, while at the same time remaining indifferent to or even favouring infection by X4 strains. Furthermore, visfatin exerts a direct effect on the relative fitness of R5 versus X4 infections in a viral competition setup. Direct interaction of visfatin with the CCR5 receptor is proposed as a putative mechanism for this differential effect. Possible in vivo relevance of visfatin induction is illustrated by its association with the dominance of CXCR4-using HIV in the plasma.

**Conclusions:**

As an innate factor produced by monocytes, visfatin is capable of inhibiting infections by R5 but not X4 strains, reflecting a potential selective pressure against R5 viruses.

## Introduction

### Background

Virus-host interactions in Human Immunodeficiency Virus (HIV) infection are characterised by a continuous arms race between the immune system and the virus. HIV is targeted by an immune response from the very first weeks of the acute phase, and the resulting immune pressure drives viral evolution throughout the infection. This diversifying selection manifests prominently (though certainly not exclusively) at the level of the variable regions of the envelope (Env) gene [1]. One example of such diversifying evolution of Env is the development of the so-called coreceptor switch, which entails the change in preferential coreceptor usage (determined by the V3 region of Env [2]) of the virus for CCR5 to CXCR4 in ∼50% of all HIV subtype B infected patients. The switch is associated with increased rates of T lymphocyte depletion and rapid progression to AIDS. Nevertheless, the viral and immune parameters underpinning this switch remain a matter of debate [3], [4].

Most studies concerning immune pressure on HIV have focused on either the adaptive cellular or the humoral arm of the immune system. Increasingly, however, the important influence of restriction factors and innate immunity on viral infectivity is recognised. Viral evolution driven by cellular responses typically hinges on viral escape from cytotoxic T lymphocyte (CTL) activity by introducing mutations in epitopes recognised by Human Leukocyte Antigen (HLA) class I molecules [5], [6], [7]. Humoral responses on the other hand result in neutralising antibodies targeted at Env epitopes, which drive the virus to rapid mutation of Env to escape this neutralising activity [8], [9]. A possible contribution of cells of mononuclear phagocyte lineage, such as monocytes and macrophages, to selective pressure on HIV infection is much less well documented. Nevertheless, in vivo and in vitro infection of macrophages by HIV has been extensively documented since the early days of the HIV pandemic [10], [11], and while they exhibit a marked resistance to the cytopathic effects of HIV which occur in T lymphocytes [12], a wealth of macrophage dysfunctions contributing to HIV pathogenesis have been described[13]. In particular, monocytes/macrophages represent one of the major viral reservoirs during infection [14], due to their longevity [15], [16], their extended phase of viral productivity [17] and the relative ease by which they disseminate HIV to other cells [18]. As a primary reservoir for HIV and as major effectors of cellular immunity, macrophages are thus uniquely placed to exert an evolutionary pressure on viral development, in addition to the humoral and cellular arms of the immune system.

A better understanding of the natural selective pressure on the virus during infection may shed light on the variability in immune control between individual patients. Specifically for a poorly understood phenomenon as the coreceptor switch, identification of novel factors involved in the immune pressure on HIV could contribute to the fine-tuning of dynamical models currently under development (e.g. [19]), in addition playing a potential role as e.g. predictive biomarkers. As mentioned above, cells of monocyte/macrophage lineage seem to be interesting and hitherto incompletely explored candidates as a source of such factors.

In a previous transcriptome analysis comparing monocytes of therapy-naïve HIV patients and healthy control donors, we demonstrated an increase in both visfatin plasma levels and visfatin monocyte mRNA and protein expression in HIV patients [20]. Additionally, we described the inhibitory effect of visfatin on infections by the lab strain HIV_BaL_ and the biological clones HIV_968-2_ and HIV_968-3_, all of which are R5 strains. As leukocytes have since been shown to represent a major source of active visfatin in other models of pathology and the documented increase may thus be of physiological relevance [21], we evaluate here whether visfatin is equally capable of inhibiting other strains of HIV, including X4 viruses, in the interest of further characterising the potential immune pressure exerted by this novel antiviral factor.

## Methods

### Ethics Statement

The study was approved by the Institutional Review Board of the Institute of Tropical Medicine and written informed consent was obtained from all donors.

### Sample Collection

20 ml blood samples were collected in EDTA-tubes from 22 therapy-naïve HIV subtype B seropositive outpatients from the HIV Clinic of the Institute of Tropical Medicine in Antwerp, Belgium. Peripheral blood mononuclear cells (PBMC) were separated via a Lymphoprep (Axis Shield, Dundee, United Kingdom) gradient and plasma was concomitantly aspirated and stored at −80°C. Monocytes were purified from the PBMC fraction using the negative selection-based Monocyte Isolation Kit II from Miltenyi-Biotec (Bergisch Gladbach, Germany), according to the manufacturer’s instructions. Yields were minimally 2×10^6^ monocytes with a purity > 85%, as verified through flow cytometry. For RNA extraction, monocytes were immediately lysed in Trizol (Invitrogen, Carlsbad, CA, USA), and Trizol pellets were stored at −80°C.

### RNA Isolation and Real-time Semi-quantitative PCR

Total RNA was prepared from the Trizol pellets by chloroform extraction, as per the manufacturer’s recommendations. Ten randomly selected samples were checked for integrity on a BioAnalyzer (BioRad, Hercules, CA, USA): no protein contamination or degradation of RNA was detected. mRNA expression of visfatin, normalised to the expression of the housekeeping gene GAPDH, was analysed using real-time semi-quantitative PCR (RT-qPCR) as described previously [20].

### Cell Isolation

For in vitro infection experiments, monocytes were isolated from PBMC obtained from buffy coats of healthy donors of the Blood Transfusion Center of Antwerp by counterflow elutriation, as described previously [22]. These cells were then differentiated to monocyte-derived macrophages (MDM) during 7 days in RPMI 1640 medium (Bio-Whittaker, Verviers, Belgium) supplemented with 10% bovine foetal calf serum (FCS; Biochrom, Berlin, Germany), penicillin (100 U/ml) and streptomycin (100 µg/ml) (Roche) and 40 ng/ml M-CSF (PeproTech, London, United Kingdom) at 37°C and 5.0% CO_2_. Half of the medium was replaced after 4 days of culture. Cells were harvested and used for experiments in the same medium (without M-CSF). All experiments were repeated with cells from six different donors. Recombinant visfatin was obtained from PeproTech. The recombinant protein batches contained < 0.01 ng/µg LPS, as assessed by quantitative chromogenic limulus amoebocyte lysate assay (QLAL) (Bio-Whittaker). Viability of cells treated with recombinant visfatin was evaluated using the cell proliferation agent WST-1 (Roche) according to the manufacturer’s instructions: no appreciable effect on cell viability was found at the concentrations used (data not shown). Production of endogenous visfatin in MDM and PBMC cultures at the RNA and protein level was not found to be modulated by in vitro HIV infection (data not shown).

### Viral Replication

For viral replication experiments, MDM or resting PBMC were plated in 96-well plates at 7.5×10^5^ cells/ml and pre-treated with recombinant visfatin (200 ng/ml) or with medium alone for control cultures for 24 hours at 37°C and 5.0% CO_2_. Then, virus was added as dilution series in sixfold and incubated for 2 hours (24 hours for resting PBMC), again at 37°C and 5.0% CO_2_. Cells were then washed 3x to remove unbound virus and incubated for 14 days (in the presence of IL2 (5 ng/ml) (Roche) for resting PBMC to rescue the infection). Productive infection was monitored via an in-house developed p24 antigen ELISA, as described elsewhere [23]. Viral infection was quantified as the TCID50 (50% tissue culture infectious dose) value, which was calculated by the method of Reed & Muench [24]. Viral infection in untreated cultures is documented in table 1 for reference purposes.

**Table 1:** Mean infectivity of HIV strains in untreated MDM and PBMC.

Coreceptor usage	HIV strain	log TCID5/ml (MDM)	log TCID50/ml (PBMC)
R5	BaL	4.07	6.20
R5	968-2	2.78	3.04
R5	968-3	2.90	3.28
X4	IIIB	1.06	5.10
X4	968-1	Non-infectious	2.98
X4	968-4	Non-infectious	3.11

TCID50: 50% tissue culture infectious dose; MDM: monocyte-derived macrophage; PBMC: peripheral blood mononuclear cell.

### p24 Production

For specific measurement of p24 production, cells were prepared in the same way as for viral replication experiments. Virus was added in sixfold at an adjusted MOI of 0.1 (titrated individually for untreated and treated cells, i.e. with a viral dose adapted to the specific treatment of the cells) and incubated for 2 hours (24 hours for resting PBMC), again at 37°C and 5.0% CO_2_. p24 production was quantified via an in-house developed p24 antigen ELISA, as described elsewhere [23].

### Time of Addition Infection Experiments

For time of addition experiments, the MT-2 setup published elsewhere was used [25]. MT-2 cells were cultured in RPMI 1640 medium (Bio-Whittaker) supplemented with 10% FCS (Biochrom), penicillin (100 U/ml) and streptomycin (100 µg/ml) (Roche). 10^4^ MT-2 cells were seeded and infected with HIV_BaL_ and HIV_968-2_ at a 0.01 MOI. Cells were incubated with the virus for the indicated times at 37°C and 5.0% CO_2_ before addition of recombinant visfatin (to a final concentration of 200 ng/ml) or medium alone for the control cultures. Cells were then incubated a further two hours at 37°C and 5.0% CO_2_ and were then washed to remove visfatin and free virus. Fresh medium was added and cultures were incubated for four days at 37°C and 5.0% CO_2_ before assessment of p24 production by ELISA [23].

### Dose-response Infection Experiments

For dose-response experiments, MDM were prepared in the same way as for viral replication experiments – pre-treatment with visfatin was done with the indicated concentrations (range 0.02-200 ng/ml) or with medium alone for control cultures. Virus was added in sixfold at a MOI of 0.1 (virus titrated on untreated cells) and p24 production was quantified via an in-house developed p24 antigen ELISA, as described elsewhere [23].

### Viral Competition Assay

To assess the relative fitness, defined as the potential for viral reproduction within a given environment, of isogenic viruses differing only in their Env sequences, in this context to compare the fitness of isogenic R5 versus X4 viruses, an assay was developed using fluorescently tagged (eGFP/DsRedExpress) replication-competent chimeric viral constructs. Existing replication-competent constructs based on the HIV_NL43_ backbone, with the eGFP or DsRedExpress sequence in concatenation with an internal ribosome entry site, were a kind gift by Dr. Kevin Ariën and Dr. Bruno Verhasselt (UGent) [26]. Through molecular cloning, the HIV_NL43_ Env sequences in these constructs were replaced with a restriction (linearization) site. Following linearization, amplified Env sequences of interest (HIV_BaL_, HIV_HxB2_, HIV_943-1_, HIV_943-3_) were cloned into the constructs using InFusion (Clontech, Mountain View, CA, USA) technology.

In this fashion, plasmids for four eGFP-labelled and four DsRedExpress-labelled chimeric viral constructs were generated (HIV_BaL_, HIV_HxB2_, HIV_943-1_, HIV_943-3_). Infectious viruses were generated by transfection of HEK293 T cells [27], as described elsewhere [28]. Since productive infections with these viruses could be established in the Jurkat [29] and SupT1[30] T lymphocyte cell lines but not in primary cell cultures, all competition experiments were performed in the Jurkat T cell line.

Infections were performed by plating Jurkat T cells in 48-well plates at 0.5×10^6^ cells/ml and 200 µl/well in RPMI 1640 medium (Bio-Whittaker) supplemented with 10% FCS (Biochrom), penicillin (100 U/ml) and streptomycin (100 µg/ml) (Roche), 50 µg/ml L-glutamine (Lonza, Basel, Switzerland) and 0.2 mg/ml geneticin (Invitrogen), adding infectious viruses at a multiplicity of infection (MOI) of 0.1, either as a mono- or as a double infection, to a final volume of 400 µl/well and incubating for 24 hours at 37°C and 5.0% CO_2_. Cells were then washed 3x to remove unbound virus and incubated further. At day 3, 7, 10 and 14 post-infection, 200 µl cell suspension was harvested for flow cytometric analysis and the volume was replenished to 400 µl. At day 7, after harvesting, the cultures were supplemented with either visfatin (200 ng/ml), maraviroc (5 nM) or normal medium as control. With each subsequent replenishment of the medium, these concentrations were maintained.

**Figure 1:**
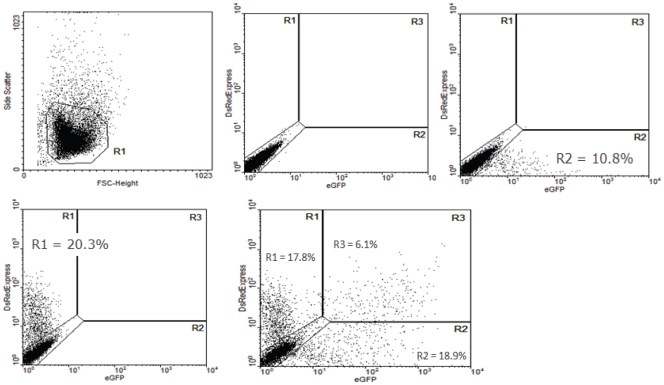
Optimization of relative fitness calculations for chimeric virus competitions. Infection of untreated Jurkat cells with BaL- and HxB2-Env-supplemented pBRNL43 IRES eGFP/DsRedExpress constructs; fluorescence levels were assessed by flow cytometry after 7 days of infection. A) Live gate; B) regions of eGFP+, DsRedExpress+ and double+; C) eGFP+ cells in BaL mono-infected cultures; D) DsRedEpress+ cells in HxB2 mono-infected cultures; E) eGFP+, DsRedExpress+ and double+ cells in BaL/HxB2 double-infected cultures.

An example of the calculation of the relative R5 versus X4 fitness is provided in figure 1 for the competition between an eGFP labelled BaL-derived and a DsRedExpress labelled HxB2-derived virus. This example documents the calculation at day 7 post infection – all calculations were done in the same way for each individual time point. The total number of fluorescent-positive cells (eGFP or DsRedExpress) for a specific virus in the double infections was normalised by the number of fluorescent-positive cells in the mono-infection of that virus, establishing the overall viral infection rate in the double infections (equations A–B). The ratio of the R5 over the X4 viral infection rate was taken as the relative R5 versus X4 fitness (equation C). All experiments were conducted in a colour-flipped setup (i.e. R5-eGFP and X4 DsRedExpress in parallel with R5-DsRedExpress and X4-eGFP) to avoid any potential bias introduced by the fluorescent labels.
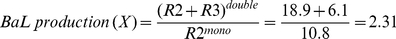
(A)

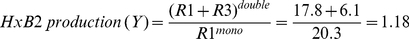
(B)

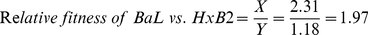
(C)


### Interaction of Visfatin with CCR5

Interaction of visfatin with CCR5 was assessed by Surface Plasmon Resonance (SPR) using a T100 Biacore instrument (GE Healthcare, Buckinghamshire, UK). The setup consisted of a CM5 chip on which an antibody raised against the 5His tag (Qiagen, Valencia, CA, USA) was immobilized, using amine coupling chemistry. This antibody (∼10000 Resonance Units) could capture ∼5000 RU of recombinant His-tagged insect-cell expressed CCR5[31], solubilized from membranes using a detergent mixture of DDM, CHAPS, CHS, and DOPC[32] at pH 7: this solution was injected over a surface constituted of amine-coupled anti-His-tag antibody. The capture of CCR5 was thus achieved through a 5His tag on its C-terminal (the intracellular part of the receptor). In this setup, CCR5 was recognized by the conformation-dependent 2D7 antibody (mouse anti-human CD195, BD Pharmingen, Franklin Lakes, NJ, USA), which recognizes well-folded (not denatured) ECL2 on the extra-cellular side (data not shown). The approach is conceptually similar to that discussed in [33].

In this setup, CCR5 was shown to be able to bind the conformation-dependent antibody 2D7 (mouse anti-human CD195, BD Pharmingen, Franklin Lakes, NJ, USA; data not shown). Experiments with visfatin (Peprotech) were performed at least twice using a flow rate of 50 µL/min during an association phase of 360 s. The detergent mixture (DDM, CHAPS, CHS, and DOPC) was present at all steps of the experiment; before, during and after visfatin injection. Signals were processed with the Biacore T100 Evaluation Software using double referencing with both a reference channel (without CCR5) and blank injections (buffer only).

### Viral Isolation and Coreceptor Usage Determination

Coreceptor usage of clinical viral isolates was determined by in silico prediction algorithms based on Env V3 loop sequences. Plasma separated from patient blood samples was stored at −80°C until use. Viral RNA was extracted from 140 µl of patient plasma using a QIAamp Viral RNA Mini Kit (Qiagen) according to the manufacturer’s instructions. Reverse transcription was performed using Expand RT enzyme (Roche Diagnostics, Mannheim, Germany) and the 3INN2b primer (5′-GTGTGTAGTTYTGCCARTCAGGG-3′). A V3-containing fragment of the Env gene was amplified from this viral cDNA and the V3 loop was sequenced using the H1E100 (5′-CGGAATTCAGIACAGTACAATGTACACATGG-3′) primer. Sequences were analysed using the Support Vector Machines (SVM) and Charge Rule algorithms for coreceptor usage prediction (available at http://genomiac2.ucsd.edu:8080/wetcat/v3.html) [34]. For a limited subset of samples, predicted coreceptor usage was confirmed by culturing the dominant virus from plasma samples and performing U373.CCR5 and U373.CXCR4 infection assays.

### Statistical Analysis

Significance of patient data was assessed via nonparametric Mann-Whitney test. All statistical calculations, including IC50 determination through nonlinear regression analysis, were performed using GraphPad Prism version 4.01 for Windows (GraphPad Software, San Diego, CA, USA). All data are expressed as mean ± SEM and representative data of at least three independent experiments is shown, except where indicated.

## Results

### Visfatin Differentially Affects Infection/Production by R5 and X4 HIV Strains

**Figure 2:**
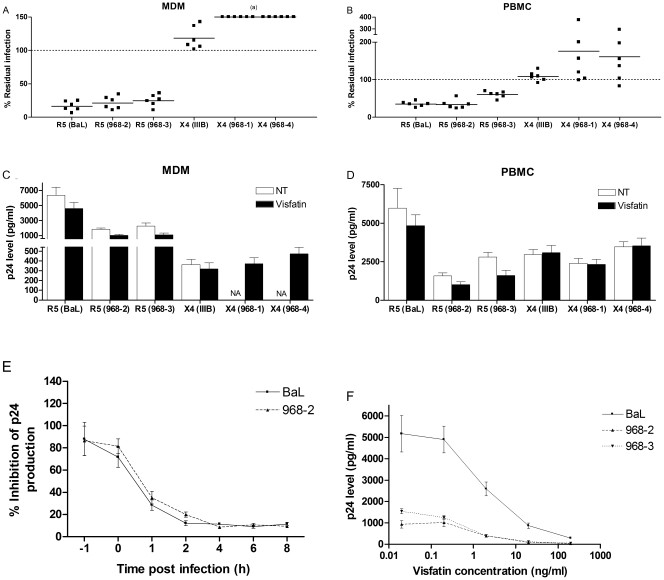
Modulation of viral infection and production of R5 and X4 HIV strains by visfatin. Infection of MDM and resting PBMC (pre-)treated with recombinant visfatin (200 ng/ml) by R5 HIV_BaL_, HIV_968-2_ and HIV_968-3_ and X4 HIV_IIIB_, HIV_968-1_ and HIV_968-4_: A-B) TCID50 values were determined using the method of Reed & Muench[24], based on p24 measurement in culture supernatants of respectively MDM and PBMC. Infection in visfatin treated cells is expressed as a percentage of infection in untreated control cells from the same donor (a): an infection could not be established in absence of visfatin treatment; results for six independent donors are shown; C-D) viral production, as quantified by p24 secretion, 14 days after respectively MDM and PBMC infection with the different strains at 0.1 MOI; means ± SEM of six independent donors are shown; E) time of addition: MT-2 cells were infected with R5 HIV_BaL_ and HIV_968-2_ and were treated with visfatin (200 ng/ml) after the indicated times; p24 production was assessed after 4 days of infection; F) viral production, as quantified by p24 secretion, 14 days after MDM infection with R5 HIV_BaL_, HIV_968-2_ and HIV_968-3_ at 0.1 MOI, in the presence of different doses of visfatin (dose-response curve). NT: untreated control.

Previously, the inhibitory effect of visfatin on infection of MDM and resting PBMC by the lab strain HIV_BaL_ and the biological clones HIV_968-2_ and HIV_968-3_ was described [20]. To evaluate the potential of visfatin to contribute to the immune control of different viral strains, parallel cultures of MDM and resting PBMC were infected with the lab strains HIV_BaL_ (R5) and HIV_IIIB_ (X4) and the biological clones HIV_968-2_ – HIV_968-3_ (R5) and HIV_968-1_ – HIV_968-4_ (X4). Viral production in visfatin-treated cells was compared to that in untreated cells from the same donor (taken as 100% production), i.e. each infection experiment was performed with its own internal control. Infection of MDM and PBMC by the R5 strains was inhibited by treatment of the cells with visfatin, as described previously: TCID50 values of R5 strains in MDM and PBMC were reduced by approximately 1 log and 0.85 log respectively in the presence of visfatin, indicating a ∼90% and ∼75% reduction respectively (fig. 2A-B).

Additionally, we measured p24 production by visfatin-treated and untreated cells after infection. Cell cultures were infected at an adjusted MOI of 0.1, i.e. titrations were done individually for both cell types and in presence and absence of visfatin – the viral dose giving 0.1 MOI in each specific setting was then used. In visfatin-treated cells in which an R5 infection was established (at an adjusted MOI of 0.1) the p24 levels were significantly lower than in untreated cells, albeit to a lesser degree than the inhibition of infection (fig. 2C-D). These data suggest that visfatin may exert inhibitory effects at two levels: inhibition of infection as such (possibly at entry level), and inhibition more downstream of entry.

Time of addition experiments in the MT-2 cell-line were performed to verify whether the kinetics of visfatin inhibition are in line with those of a viral entry inhibitor. The rapid reduction in inhibition when visfatin was added to infected cultures after one hour or more is suggestive of entry inhibition, though inhibition was not abrogated completely (fig. 2E), again suggesting a possible dual effect of visfatin. To correctly assess the inhibitory effect of visfatin, and to ascertain that the doses needed to establish inhibition in vitro are compatible with reported serum visfatin loads of 1-10 ng/ml in vivo[35], a dose-response curve was established for the HIV_968-3_ strain (fig. 2G) – p24 levels of untreated control cells were in the range of those in cells treated with the lowest dose of visfatin (0.02 ng/ml – data not shown). Based on these dose-response curves, the EC50 values of visfatin in MDM were calculated (table 2).

**Table 2:** IC50 values of visfatin in MDM for R5 HIV strains.

HIV strain	IC50 (95% CI)
BaL	1.24 ng/ml (1.15–1.38)
968-2	1.46 ng/ml (0.44–4.95)
968-3	1.15 ng/ml (0.46–1.12)

IC50: 50% inhibitory concentration.

Interestingly, this inhibitory effect was not observed in cells infected with the X4 strains HIV_IIIB_, HIV_968-1_ and HIV_968-4_. Infection by HIV_IIIB_ and production of p24 in presence or absence of visfatin was not affected. Effects on the X4 clones 968-1 and 968-4 were even more pronounced: these clones were not capable of infecting untreated MDM but could establish a productive infection in MDM after visfatin treatment (fig. 2A&C). This differential effect was also observed in PBMC, where visfatin did not inhibit, and in some donors even augmented, infection by X4 HIV_968-1_ and HIV_968-4_. However, in cells which were infected (adjusted MOI of 0.1 for treated and untreated cells), p24 production was not modulated by visfatin treatment (fig. 2B&D). Thus, visfatin appears to be capable of applying differential inhibitory activity on different strains of HIV, seemingly dependent on the coreceptor usage of these strains.

### Visfatin Exerts a Selective Pressure on HIV Favouring X4 Strains

The differential effect of visfatin on R5 versus X4 HIV strains suggests that it may contribute to a general “R5 unfavourable” environment, favouring development of X4 strains. However, these observations were based on infection assays using viruses which differ in more than merely their coreceptor usage. Additionally, the observed effects in single viral infections do not necessarily confer a fitness advantage of X4 over R5 viruses, as effects may be too slight to be of in vivo relevance and the complexity of viral dynamics in a competitive setup is not accounted for. We therefore developed an assay for the evaluation of the relative fitness, defined as the potential for viral reproduction within a given environment, of quasi-isogenic R5 and X4 viruses, using fluorescently tagged (eGFP/DsRedExpress) replication-competent chimeric viral constructs into which Env sequences of interest (R5 and X4 viruses) were cloned.

**Figure 3:**
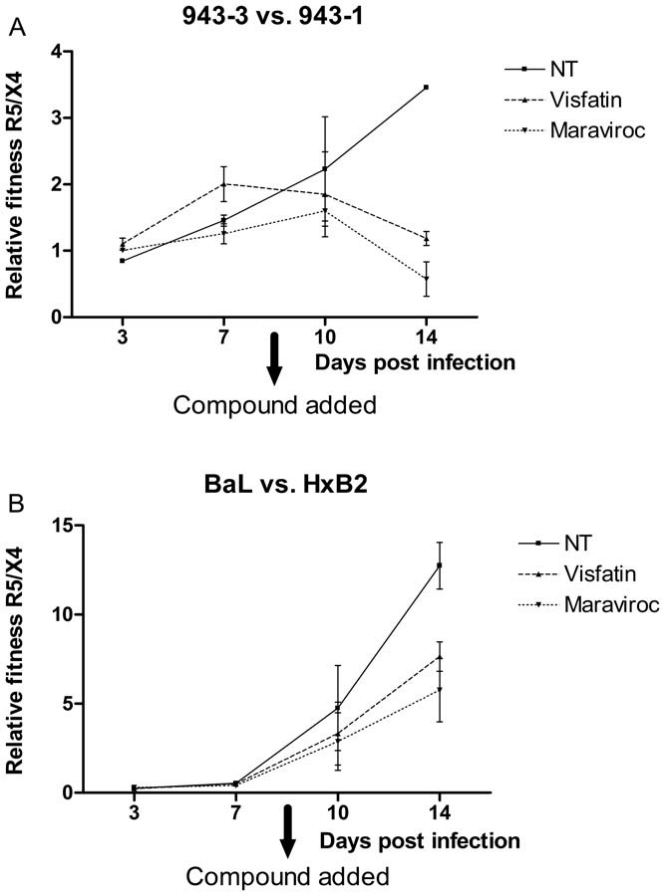
Relative fitness of R5 versus X4 HIV strains in presence and absence of visfatin and maraviroc. Flow cytometric evaluation of infection rates of Jurkat T cells by eGFP and DsRedExpress chimeric viruses at 0.1 MOI in presence and absence of visfatin (200 ng/ml) or maraviroc (5 nM): A) HIV_943-3_ (R5) versus HIV_943-1_ (X4) and B) HIV_BaL_ (R5) versus HIV_HxB2_ (X4). Ratios are depicted of infection rates in double infections normalised to the mono-infection rates; all infections were performed in a colour-flipped setup. Mean and SEM were calculated based on the colour-flipped datasets within each experiment; representative results for three independent experiments are shown. NT: untreated control.

Here, Env sequences of the lab strains HIV_BaL_ (R5) and HIV_HxB2_ (X4) and the clinical isolates HIV_943-3_ (R5) and HIV_943-1_ (X4) were used to complement these chimeric viral constructs, which were competed against each other in Jurkat T cell line cultures. The clinical isolates were selected for their Env sequence similarity (as they were isolated from the same patient) and their relatively matched fitness [36]. After 7 days of competition, visfatin or the CCR5 antagonist maraviroc (as positive control) were added to the cultures to evaluate whether they could modulate the relative fitness of the R5 versus the X4 strains. In all competitions (Env sequences of lab-attenuated strains and of clinical isolates), a clear effect on the relative fitness was observed: R5 versus X4 relative fitness decreased as soon as visfatin or maraviroc were added (fig. 3). In the competitions involving Env sequences from clinical isolates, effects were most pronounced, with viruses complemented with X4 Env exhibiting dominance in the cultures after visfatin treatment (fig. 3A). For the competitions involving the lab-attenuated strains, a significant reduction in the relative fitness of R5 over X4 viruses was observed, though the R5 viruses maintained their dominance (fig. 3B). No significant additive effects of visfatin on maraviroc were observed (data not shown). Visfatin therefore seems capable of directly exerting a selective pressure against R5 and favouring X4 viruses.

### Visfatin Interacts Directly with the CCR5 Coreceptor

**Figure 4:**
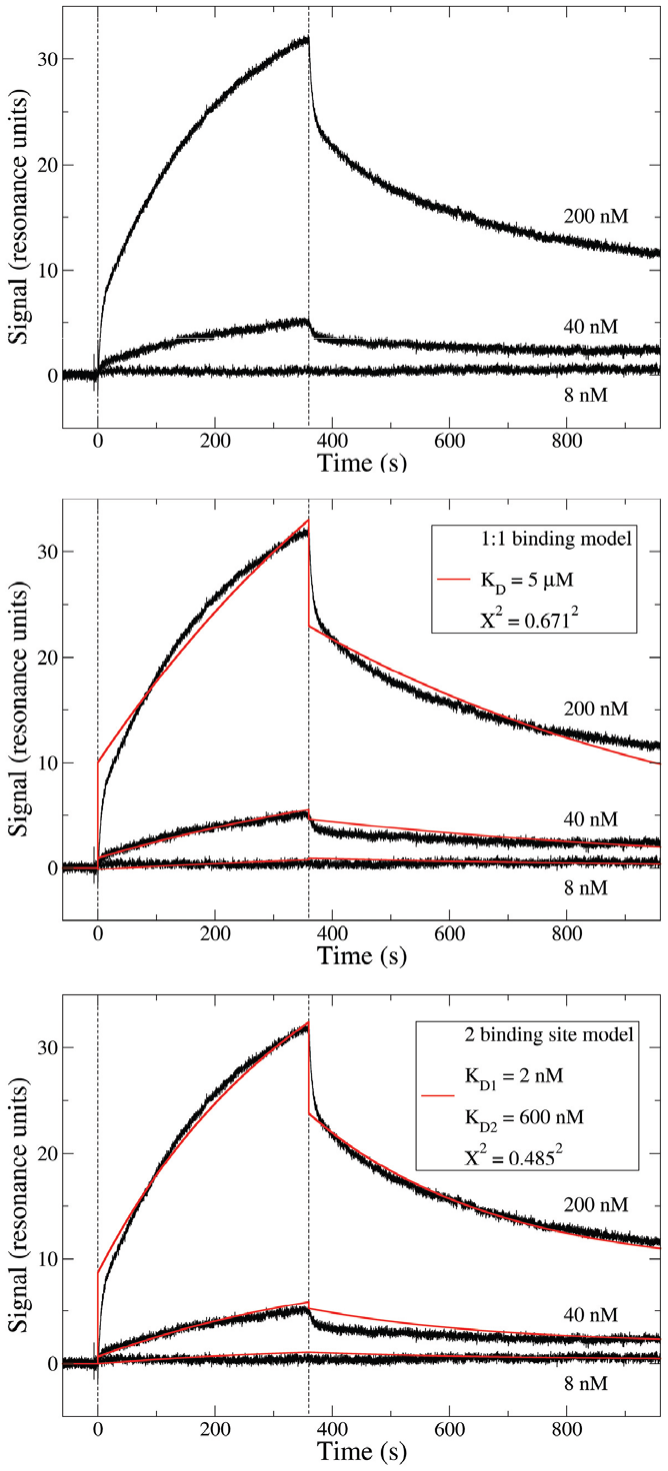
Characterisation of visfatin-CCR5 interaction, as assessed by SPR. Different concentrations of visfatin were injected sequentially on immobilized CCR5. Vertical dashed lines indicate the start and end of injection. A) Raw data; B) fitting to a 1∶1 binding model and C) to a two binding site model, with respective K_D_ and X^2^ values.

Previously, we demonstrated that visfatin acts on early, pre-integration events in the R5 viral life cycle, as evidenced by the reduced integration of proviral DNA in infected cells in the presence of visfatin [20]. However, we were not able to identify the inhibitory mechanism underpinning the observed visfatin activity. In view of the differential effects observed here, we evaluated whether visfatin could interact directly with the CCR5 coreceptor, which could provide a mechanistic basis for the observed inhibition of R5 infection. Binding studies between visfatin and detergent-solubilised CCR5 were conducted in a SPR setup. A specific interaction between visfatin and CCR5 was observed (fig. 4). In accordance with the functional dimerisation of visfatin, a two-binding site model resulted in an improved fitting of the curve (as opposed to a 1∶1 binding model, fig. 4B-C) and suggested that the affinity of visfatin for CCR5 was in the nM range, whereas the K_D_ for the dimerisation would be in the µM range. Consistent with the hypothesis of visfatin dimerisation on CCR5 after the binding of the first visfatin monomer, the fitted populations for both binding sites were approximately 50% each.

### Visfatin Expression Correlates with Coreceptor Usage of Clinical Isolates

In order to establish whether clinical observations support the in vitro observation that visfatin contributes to an “X4 favourable” environment, the coreceptor usage of dominant plasma viruses in therapy-naïve HIV patients was evaluated. Coreceptor usage was assessed using in silico prediction algorithms based on the sequence of the variable V3 loop of the HIV Env gene [34]. Out of the 22 samples assessed, six patients turned out to harbour X4 viruses. For three of these patient plasma samples, viral culturing was possible and viral coreceptor usage was confirmed by infection experiments of CCR5- or CXCR4-expressing U373 cells. These samples were matched for age, CD4 T Lymphocyte count and viral load with R5 samples, again three of which were confirmed as R5 by in vitro infection experiments.

**Figure 5:**
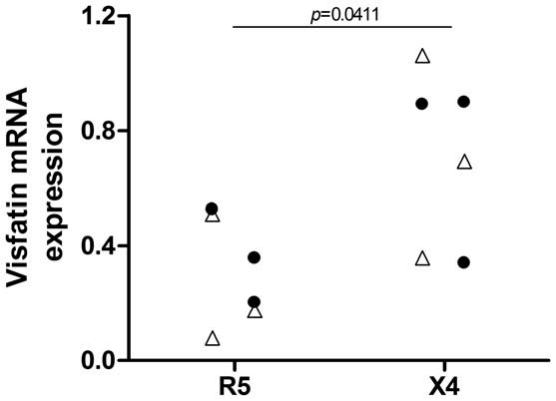
In vivo association of visfatin expression with coreceptor usage. mRNA expression of visfatin in monocytes of therapy-naïve HIV patients, normalised to GAPDH expression, as assessed by RT-qPCR; patients were grouped according to the coreceptor usage of the dominant plasma viral isolates, as assessed by in silico prediction algorithms (Support Vector Machines and Charge Rule algorithms) (triangles) or both in silico algorithms and U373.CCR5/CXCR4 infection assays (filled circles) and were matched for age, CD4 T Lymphocyte count and viral load. *p*-value calculated by nonparametric Mann-Whitney test.

Visfatin monocyte mRNA expression, as quantified by real-time qPCR, was compared between these two groups. When patients were grouped according to the coreceptor usage of their corresponding plasma viruses, clear differences were found between the groups at the level of visfatin mRNA expression. High visfatin expression appeared to correlate with the dominance of X4 viruses, while low visfatin expression was associated with R5 viruses (fig. 5). As these groups were matched for viral load, the observed difference cannot be due to higher visfatin expression resulting from high viral loads in patients having undergone the coreceptor switch. This correlation reinforces the notion that visfatin can contribute to an environment which is more favourable towards X4 HIV than R5.

## Discussion

In this study, we further characterised the factor visfatin, which was found to be induced in monocytes of therapy-naïve HIV patients in a previous transcriptome analysis [20]. As we initially described visfatin as a putative innate antiviral factor induced during HIV infection based on infections with R5 strains of HIV, we evaluated the effects of visfatin on MDM and PBMC infection by X4 strains of HIV, in order to ascertain whether visfatin can modulate general HIV infection. Our observations suggested that visfatin can exert a selective pressure on HIV, in that it is capable of inhibiting infection by R5 strains of HIV but not X4 HIV strains. The kinetics of visfatin-mediated R5 HIV inhibition suggest that visfatin may act on the early steps in the viral life cycle, such as viral entry into the cell.

Furthermore, p24 production in R5 HIV-infected MDM and PBMC appeared to be modulated by visfatin treatment when treated and untreated cells were infected at the same MOI (adjusted to compensate for the reduction in infectivity under treatment). This suggests that there might be further inhibitory effects of visfatin downstream of entry, but occurring upstream of integration of viral DNA in the host genome (as discussed in [20]). Interestingly, this effect on reduction of p24 expression was not observed in X4 HIV-infected cells, which may reflect differences in post-entry replication stages between R5 and X4 viruses [37], [38], [39]. Direct competition experiments indicated that visfatin does indeed modulate the relative fitness of R5 versus X4 viral strains in vitro. This notion was further supported by the clinical observation that high visfatin expression is associated with dominance of X4 HIV in the plasma, rather than R5.

These data suggest that visfatin can indeed to some extent modulate the immune environment of the virus. Whether these in vitro results can be translated to in vivo effects and whether visfatin is directly involved in e.g. the coreceptor switch remains to be elucidated. Evidently, the coreceptor switch cannot be attributed to any individual factor (such as visfatin). Nevertheless, visfatin could represent at least a peripheral parameter in the coreceptor switch by facilitating X4 selection over R5 through effects on the immune environment, in a fashion analogous to the documented selection of X4 over R5 viruses during treatment with CCR5 inhibitors[40]. As such, it could constitute an additional parameter in the different mathematical models of the coreceptor switch currently being constructed [19], [41], [42].

Previously, we reported that visfatin may act on early events of the viral life cycle, as the integration of proviral DNA is abrogated by visfatin treatment, and that reduced binding of R5 HIV to the cell membrane would be a plausible mechanism for this inhibitory activity [20]. In this study, we demonstrated the direct binding of visfatin to the CCR5 receptor, suggesting a mechanism for inhibition of R5 but not X4 viral binding to the cell. Additionally, the finding that visfatin binds directly to the CCR5 receptor may shed light on the continuing controversy on the visfatin-receptor interactions (e.g. [43]), and should be evaluated in the context of the plethora of immune activities ascribed to this factor [44].

In conclusion, we suggest that the adipocytokine visfatin acts as a novel monocyte-derived innate factor capable of deterring HIV infection. As such, it represents one of the many host-derived factors capable of exerting immune pressure on the virus. In particular, the discriminatory activity of visfatin favouring X4 and hampering R5 HIV may be of relevance in the HIV coreceptor switch, for which it may at the very least represent a promising candidate as biomarker.
